# Efficacy and safety of polydioxanone thread embedded at specific acupoints for non-specific chronic neck pain: a study protocol for a randomized, subject-assessor-blinded, sham-controlled pilot trial

**DOI:** 10.1186/s13063-018-3058-9

**Published:** 2018-12-06

**Authors:** Eunseok Kim, Hye Su Kim, So-Young Jung, Chang Hyun Han, Young-Il Kim

**Affiliations:** 1 Department of Acupuncture and Moxibustion Medicine, College of Korean Medicine, Daejeon University, 62, Daehak-ro, Dong-gu, Daejeon, 34520 Republic of Korea; 2 Department of Acupuncture and Moxibustion Medicine, Dunsan Korean Medicine Hospital of Daejeon University, 75, Daedeok-daero 176 beon-gil, Seo-gu, Daejeon, 35235 Republic of Korea; 3 Clinical Research Division, Korea Institute of Oriental Medicine, 1672, Yuseongdae-ro, Yuseong-gu, Daejeon, 34054 Republic of Korea

**Keywords:** Chronic neck pain, Polydioxanone, Thread-embedding acupuncture, Efficacy, Randomized sham-controlled trial

## Abstract

**Background:**

This study aims to evaluate the efficacy and safety of thread-embedding acupuncture (TEA) with polydioxanone thread embedded at various acupoints, compared with sham TEA, for the treatment of non-specific chronic neck pain.

**Methods/design:**

This study will be an 8-week-long, two-armed, parallel, randomized, subject-assessor-blinded, sham-controlled pilot trial. Fifty eligible patients will be randomly allocated into the real TEA group or the sham TEA group. The real TEA group will receive TEA treatment at 14 fixed acupoints in the neck region. The sham TEA group will receive the same treatment as the real TEA group, but with a sham device with the thread removed. Both groups will receive treatment once a week for a total of four sessions. The primary outcome will be the mean change in the visual analog scale (VAS) from baseline to week 6 (2 weeks post intervention). Clinical relevance (ratio of the number of patients with decreases on the VAS of ≥15 mm or with percentiles ≥ 30% and ≥ 50% relative to baseline to the total number of patients), Neck Disability Index, pressure pain threshold, the Hospital Anxiety and Depression Scale, EuroQol 5-Dimensions questionnaire, Patient Global Impression of Change, blinding test, and adverse events will be used to assess secondary outcomes.

**Discussion:**

The results of this study will provide valuable data for a large-scale clinical trial to evaluate the clinical effects of polydioxanone TEA in the treatment of patients with non-specific chronic neck pain.

**Trial registration:**

Clinical Research Information Service (CRIS), Republic of Korea, KCT0002452. Registered on 6 September 2017.

**Electronic supplementary material:**

The online version of this article (10.1186/s13063-018-3058-9) contains supplementary material, which is available to authorized users.

## Background

Non-specific chronic neck pain (CNP) is a common health problem, affecting 30–50% of the general population, and 23% of those individuals with CNP develop a recurrent episode within a month after their recovery [1, 2]. Of adult Europeans, 19% have chronic pain seriously affecting their daily activities and, of those, 20% have neck pain [3]. In the United Kingdom, 29% of adults experienced back or neck pain in the prior month, with half of those reporting chronic pain [4], and these observations are similar to those on a United States survey [5]. According to a survey conducted between 2007 and 2008 in Korea, the lifetime prevalence of neck pain is 20.8% [6]. CNP is also common in most occupational groups and is related to disability in both social and occupational groups because 11.0–14.1% of workers are limited in their activities each year [1, 7–9]. The economic burden associated with neck pain is increasing because many patients tend to continue to utilize health care resources for treatment for up to 10 years after the initial onset [10, 11].

Various non-surgical treatments, such as non-steroidal anti-inflammatory drugs (NSAIDs), muscle relaxants, analgesics, physiotherapy, interdisciplinary rehabilitation, education, and spinal injection, are available for the treatment of patients with CNP [12–14]. Although pain-relieving medication is most frequently used to alleviate CNP, long-term NSAID use is limited due to the risk of side effects and patient intolerance [15, 16]. On the other hand, acupuncture has been increasingly recognized as being clinically beneficial because it has been consistently reported to be effective and safe in reducing pain and improving quality of life for patients with neck pain [13, 17–19].

Thread-embedding acupuncture (TEA) is a special type of acupuncture designed to embed thread at targeted tissue by using a hollow needle. Absorbable suture materials, such as polydioxanone (PDO), catgut, PCL (polycaprolactone), etc., are used for the threads in the TEA devices [20]. PDO thread has been used in TEA devices in Korea while catgut thread is used in China. PDO thread is known to take approximately 180 days after implantation to be completely absorbed by the tissue [21]. TEA is considered to have longer therapeutic effects through continuous stimulation by the threads embedded at the acupoints.

Randomized clinical trials (RCTs) in China evaluating the effect of catgut thread embedded at certain acupoints for the treatment of patients with various diseases, polycystic ovary syndrome, obesity, and postmenopausal period, have been reported [22–24]. A previous study also demonstrated the safety and efficacy of catgut TEA for the treatment of allergic rhinitis by using a sham device [25]. In Korea, the embedding of PDO thread at certain acupoints is widely used in the clinical practice of Korean Medicine for the treatment of chronic musculoskeletal pain. Nevertheless, the current level of evidence supporting the efficacy of embedding with PDO thread for patients with chronic musculoskeletal pain is insufficient. Only case studies on the application of PDO TEA for the treatment of patients with chronic low back pain [26], shoulder pain [27], and osteoarthritis of the knee [28] have been published. In addition, no RCTs using PDO thread embedded as a sham-controlled intervention have been reported [29].

Therefore, we designed a pilot RCT to investigate the efficacy of PDO thread embedded at certain acupoints for the treatment of patients with CNP. In addition, adverse effects of PDO TEA will be evaluated for safety assessment.

## Method/design

### Objective

The primary objective of this pilot study is to assess the therapeutic efficacy of PDO thread embedded at certain acupoints for the treatment of patients with CNP, compared to sham TEA, and to obtain valuable data and insights for a confirmative, full-scale RCT.

### Design

This study will be a single-center, equally randomized, two-armed, parallel, stratified (gender), subject-assessor-blinded, sham-controlled pilot trial. This trial is being conducted at the Pain and Spine Center, Dunsan Korean Medicine Hospital of Daejeon University (DKMHDU), Korea.

A total of 50 participants diagnosed with CNP will be recruited from the Clinical Trial Center of DKMHDU in Daejeon through advertisements in local newspapers, on hospital websites and bulletin boards, in information leaflets, etc. The recruitment period will run from May 2017 to December 2017. Clinical research coordinators will interview all potential participants over the telephone and schedule screening visits. At the screening visits, all potential participants will be informed of the objectives and the overall procedure of the trial and will be asked to provide written informed consent. Study volunteers will go through a screening process, which will include radiography of the cervical spine and physical examinations, to verify the diagnosis of non-specific CNP.

**Fig. 1:**
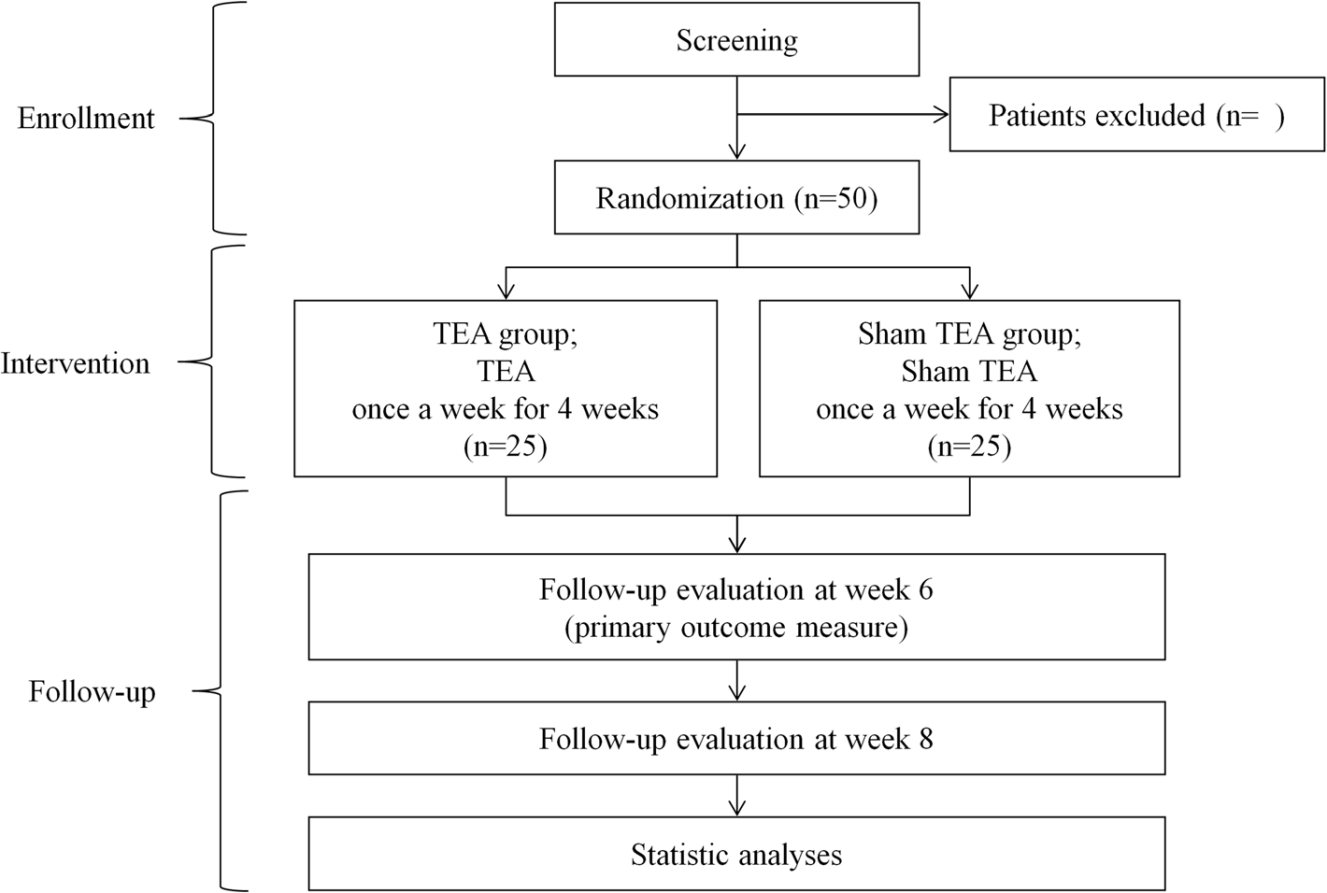
Flowchart of the study

**Fig. 2:**
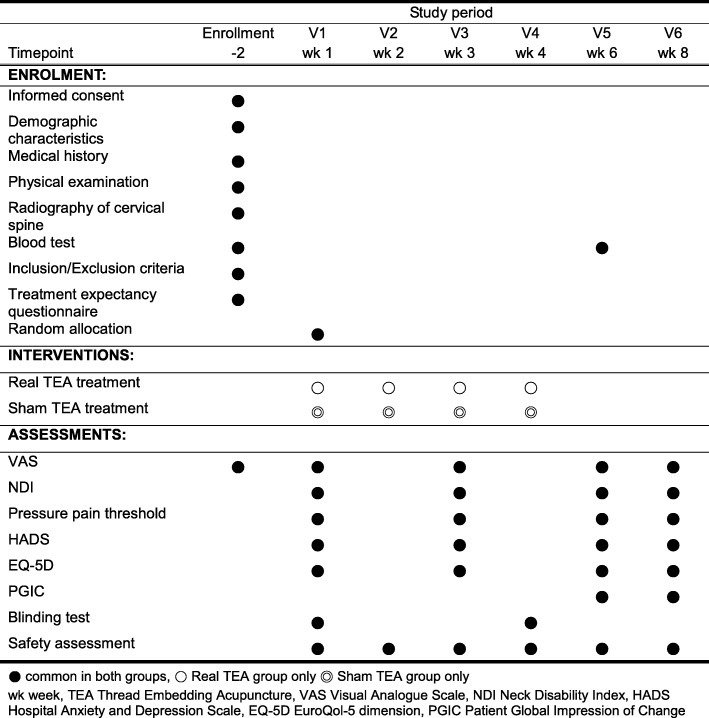
Schedule for the treatment and outcome measurements

At visit 1, an eligible participant will receive baseline assessments, including the use of the acupuncture expectancy scale [30, 31], and will then be randomly allocated into one of two groups: the real TEA group and the sham TEA group. The real TEA group will receive TEA treatment at local acupoints in the neck region. The sham TEA group will receive the same treatment as the real TEA group, but with a sham device with the thread removed. Both groups will receive treatment once a week for a total of four sessions. The real TEA and the sham TEA will be described to the participants as “classical TEA” and “non-classical TEA,” respectively. Researchers will explain to the participants that the term “classical TEA” is “typically used in Korean Medicine clinics” while the term “non-classical TEA” is “rarely used in Korean Medicine clinics” [32, 33]. Outcome measures will be assessed before the treatment (week 1), 2 weeks after the first treatment (week 4), and 2 weeks (week 6) and 4 weeks (week 8) after the last treatment. A flowchart of the study is illustrated in Fig. 1, and the clinical trial schedule is presented in Fig. 2.

### Participants

#### Inclusion criteria

A participant will be enrolled if they fulfill the following criteria: (1) age 19–65 years; (2) suffering from persistent or recurrent neck pain lasting longer than 3 months [34] (neck pain is defined as “pain, ache, or discomfort” in the area between the occiput and the third thoracic vertebra and between the medial borders of the scapulae [35]); (3) indicating pain of more than 40 mm on the visual analog scale (VAS) at the time of screening; (4) diagnosis of non-specific neck pain by a clinician based on history taking, physical examination, image examination, and medical examination; (5) able and willing to comply with the intervention and follow-up evaluation; and (6) able to provide written informed consent.

#### Exclusion criteria

Participants with any of the following conditions will be excluded: (1) radicular pain in the upper extremity with the distribution of a particular nerve root; (2) neurological abnormality: (a) weakness, paresthesia (sensory deficits), (b) positive sign on a special test: shoulder abduction relief sign test (Bakody sign), Spurling test, (c) hyporeflexia of the deep tendon reflex, (d) pathological reflex: Hoffman sign, ankle clonus, Babinski sign, (e) muscle atrophy; (3) major spinal pathology such as neoplasm, myelopathy, spondylitis, and congenital abnormality; (4) history of spinal surgery or scheduled for surgery during the study; (5) pain in another region that is more severe than the neck pain; (6) uncontrolled lumbar pain; (7) history of TEA treatment; (8) hypersensitive reaction to previous acupuncture treatment, metal allergy, keloid, severe atopy, and other skin hypersensitivities; (9) hemorrhagic disease and/or factors that can affect hemostasis, such as anti-coagulant or anti-platelet drug use; (10) pregnancy, lactation, or a plan to become pregnant during the study period; (11) uncontrolled diabetes, or major cardiovascular disease; (12) history of neurotic or major psychiatric disability or cognitive instability; (13) history of alcoholism or drug abuse; and (14) severe underlying disease requiring active therapy. Furthermore, participants considered to be inappropriate for the study by the researcher will be excluded.

#### Randomization and blinding

An independent, blinded statistician will generate the random number table by using the stratified randomization method of SAS® Analytics Pro (SAS Institute, Inc., Cary, NC, USA). The gender (male vs. female) will be applied as a condition value for the stratification. The random number table will be kept by the independent statistician and concealed with a password not to be exposed.

At visit 1, after baseline assessments, participants will be randomized into the experimental (real TEA treatment) group or the control (sham TEA treatment) group. Although the practitioner cannot be blinded, both participants and outcome assessors will be blinded to the allocation throughout the study period.

The study volunteers who have experience with TEA treatment will be excluded to prevent allocation guessing through discomforts, such as stiffness and irritation, post TEA treatment. Both the TEA treatment and the sham TEA treatment will be conducted by an individual practitioner, and identical treatment procedures in the same treatment room environments will be used. Because the sham TEA device appears identical to the real TEA device except for the thread hanging outside the needle, before starting the TEA treatment, the participants while in a sitting position will be instructed to turn their back on the practitioner to prevent them from being able to note the existence, or not, of the thread in the TEA needle.

### Interventions

All subjects will receive a total of four sessions for the real TEA or the sham TEA (dry-needling) treatment, i.e., once a week for 4 weeks. To avoid any non-specific effect caused by interactions, the practitioner will not be allowed to give any positive or negative encouragement for future progress. For proper treatment, the practitioner will be allowed to conduct minimal examination of subjects, which include palpating the subject’s neck to find the appropriate acupoints. All the treatment procedures and regimens will be described in detail in a pre-specified protocol and standard operating procedures (SOPs) to ensure that the conditions experienced by both group, except for the TEA device, are identical. Both the real TEA treatment and the sham TEA treatment will be consistently performed by the same individual practitioner throughout the study period. The practitioner has completed 6 years of education for Traditional Korean Medicine and has at least 4 years of clinical experience with TEA. The practitioner was trained in the trial protocol and proficient in TEA treatment prior to the study. Both real and sham TEA treatment comply with the Standards for Reporting Interventions in Clinical Trials of Acupuncture (STRICTA) (see Additional file 1) [36]. Co-intervention will not be allowed except for rescue medicine (acetaminophen, maximum dose 3000 mg/day). The exact dose of the rescue medicine will be recorded in the case report form at each visit.

#### PDO TEA treatment

The needle that will be used for TEA is made up of two parts: the external needle and the internal PDO thread. We will use disposable, sterile, PDO-thread-embedding devices (OV World Co., Seoul, Republic of Korea) with 29-gauge (G) needles and USP size 6–0 PDO thread (Samyang Biopham Co., Seongnam-si, Republic of Korea). Two standard sizes of needle will be used: 29-G × 38-mm needle with 54-mm PDO thread (27 mm × 2; folded in half) and 29-G × 25-mm needle with 30-mm PDO thread (15 mm × 2; folded in half). One side of the PDO thread folded in half is inside the hollow needle while the other side of the thread is hanging outside the needle with fixation by the thread anchor (polystyrene). The threads will be embedded bilaterally at local acupoints in the neck region. The acupoints are selected on the basis of *Meridian Theories*, the text book [20], preceding studies on acupuncture for patients with CNP [37, 38], and the consensus of Korean acupuncture experts. The seven TEA points used in this study are GB20, TE16, LI17, GB21, and SI14 for regular acupoints and two paravertebral points, one each at the levels of the fifth and the seventh cervical vertebrae (C5 and C7), for extra acupoints (Table 1). The paravertebral points are located at 0.5 body-*cun* lateral to the spinous process. The 29-G × 38-mm needle will be applied at LI17, GB21 and paravertebral points at C7, and the 29-G × 25-mm needle will be used for GB20, TE16, SI14, and paravertebral points at C5. The TEA needles will be removed immediately after needle insertion, and manual manipulation for *Deqi* will not be allowed. The acupoints will be sterilized with a disposable 70% isopropyl alcohol swab prior to needle insertion. All procedures will be performed according to the Clean Needle Technique [39].

**Table 1:** Locations of needling details for the acupoints used in the treatment protocol

Acupoints	Location	Needling details
GB20 (*Fengchi*)	In the posterior region of the neck, inferior to the occipital bone, in the depression between the origins of the sternocleidomastoid and the trapezius muscles	Perpendicular insertion in the direction of the opposite eye
TE16 (*Tianyou*)	In the anterior region of the neck, at the same level as the angle of the mandible, in the depression posterior to the sternocleidomastoid muscle	Oblique superior insertion across the sternocleidomastoid muscle
LI17 (*Tianding*)	On the anterior aspect of the neck, at the same level as the cricoid cartilage, just posterior to the border of the sternocleidomastoid muscle	Oblique inferior insertion along the scalene
GB21 (*Jianjing*)	In the posterior region of the neck, at the midpoint of the line connecting the spinous process of the 7th cervical vertebra (C7) with the lateral end of the acromion	Oblique medial insertion
SI14 (*Jianwaishu*)	In the upper back region, at the same level as the inferior border of the spinous process of the 1st thoracic vertebra (T1), 3 body-*cun* lateral to the posterior median line	Oblique inferior insertion in the direction of the superior part of the medial border of the scapula
Paravertebral point	0.5 body-*cun* lateral to the spinous process at the levels of the 5th and the 7th cervical vertebrae, respectively	Perpendicular insertion

#### Sham TEA treatment

**Fig. 3:**
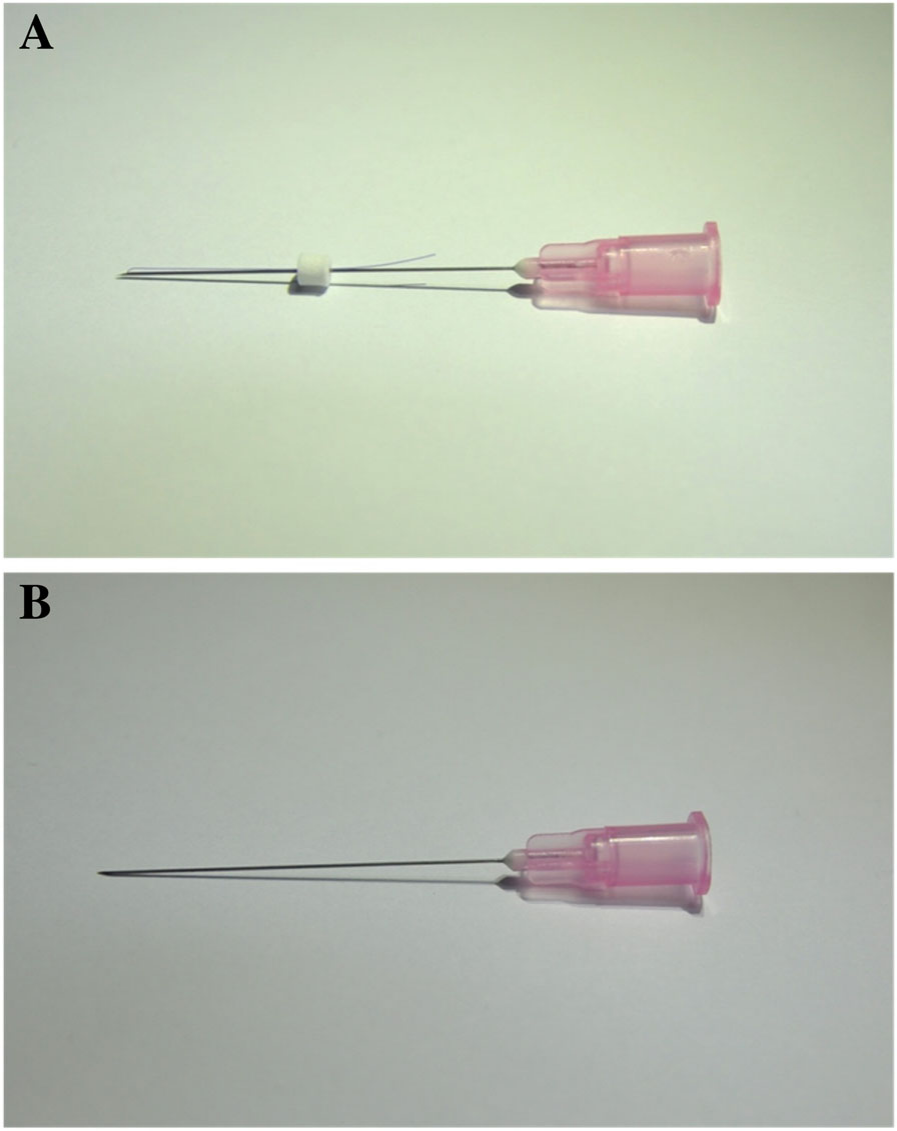
Thread-embedding acupuncture (TEA) device. **a**: needle used for real TEA. **b**: needle used for sham TEA (without polydioxanone (PDO) thread)

The sham TEA device will be a dry needle with the PDO thread removed. The sham TEA device will be made by the same manufacturer and will be, for all practical purposes, identical to the real TEA device except for the absences of the thread hanging outside the needle and the thread anchor (Fig. 3). For the sham TEA group, the entire TEA treatment procedure, such as number of acupoints, selection of needle size for each acupoint and needle-insertion method, will be the same as for the real TEA group. In other words, subjects in the control (sham TEA) group will receive only the stimulus of dry-needling, but not that of the embedded PDO thread.

### Outcome measurement

Outcome measurements will be performed before the treatment (baseline; week 1), 2 weeks after the first treatment (week 3), and 2 weeks (week 6) and 4 weeks (week 8) after the last treatment.

#### Primary outcome

The primary outcome will be the mean change on the 100-mm VAS from the baseline (week 1) to the primary endpoint (week 6), which will be 2 weeks after the completion of TEA treatment. The 100-mm VAS will be used to assess CNP because it is a fast and straightforward method for evaluating the subjective degree of pain intensity [40]. Participants will be instructed to report the intensity of neck pain experienced within the past week on a linear scale of 0 to 100 (0, no pain; 100, pain as bad as it could be) [41].

#### Secondary outcomes

The secondary outcome will consist of pain, clinical relevance, disability, pressure pain threshold, psychological stress, quality of life, global assessment, safety, and blinding test.

### Pain

The secondary outcome for pain will be the mean change on the 100-mm VAS for CNP from the baseline to week 3, week 6, and week 8, respectively.

### Clinical relevance

Clinical relevance will be assessed using the Minimal Clinically Important Difference (MCID), which is the minimal change in the evaluation score that is clinically meaningful for patients [42]. One should note that a statistically significant difference between two groups may not always be clinically significant. Several previous studies have reported the MCID on the VAS for chronic pain as 15 mm [43–45]. According to a consensus statement of the MCID recommendation, a decrease greater than 30 and 50% in the VAS score is considered as a moderate clinically important change and a substantial improvement, respectively [46]. Based on these studies, the clinical relevance for CNP will be evaluated by using the ratio of the number of patients with decreases on the VAS ≥ 15 mm or with percentiles ≥30% and ≥ 50% relative to baseline to the total number of patients, respectively.

### Disability

The Neck Disability Index (NDI) will be used to measure functional disability in the neck [47]. The validated Korean version of the NDI will be used for this trial [48]. The NDI is the most widely used questionnaire to evaluate cervical pain and dysfunction in daily life and consists of 10 questions, with a 6-point Likert scale from 0 to 5 points. The overall score ranges from 0 to 50 points, and a higher score indicates a greater degree of perceived functional disability.

### Pressure pain threshold

A Digital Pressure Algometer device (Wagner Pain Test™ – Model FPX-25 (WAGNER INSTRUMENTS, Greenwich, CT, USA)) will be used to measure the pressure pain threshold (PPT) at three points on both sides: the bilateral levator scapulae, trapezius descendens, and paravertebral point of the sixth cervical spine [49]. The PPT will be measured twice at each point, and the mean value (kg/cm^2^) will be used.

### Psychological stress

The Hospital Anxiety and Depression Scale (HADS) is a simple self-assessment scale to detect anxiety and depression in patients and has been shown to be reliable and valid [50]. The HADS consists of a total of 14 items: a seven-item depression scale and a seven-item anxiety scale. Both anxiety and depression are known to be correlated with CNP [51–53]. The Korean version of the HADS will be used for this trial [54].

### Quality of life

The Korean version of the EuroQol 5-Dimension (EQ-5D) questionnaire will be used for evaluating the quality of life (QOL) of the participants [55]. The EQ-5D consists of five categories (mobility, personal care, daily activities, pain/discomfort and anxiety/depression), and each category contains three statements describing personal health status [56]. Participants will instructed to select the most appropriate one among the three statements.

### Global assessment

The participants’ global assessment will be performed using the Patient Global Impression of Change (PGIC), a self-reported 7-point categorical scale that is used to evaluate the overall improvement after treatment [57]. Participants will evaluate themselves as to the improvement of their symptoms at week 6 and week 8 from baseline by selecting one of the following seven options: (1) very much improved, (2) much improved, (3) minimally improved, (4) no change, (5) minimally worse, (6) much worse, or (7) very much worse.

### Adverse events and safety

In this study, various post-intervention responses, such as pain, stiffness, and irritation, will be identified as adverse events related to the TEA treatment. Researchers will record the kinds of these adverse events, as well as the day that the adverse event occurs and is over. The duration of each adverse event will be recorded as a treatment outcome to compare between-group differences. Any causal relationship between the TEA treatment and adverse events will be evaluated using a 6-grade scale (1 = definitely related, 2 = probably related, 3 = possibly related, 4 = probably not related, 5 = definitely not related, and 6 = unknown), and the seriousness of the adverse events will be scored using a 4-point Spilker scale (1 = mild, 2 = moderate, 3 = severe, and 4 = extremely severe). If any serious adverse event (SAE) occurs, the researcher will immediately inform the SAE to the primary investigator (PI). The allocation status of the subject will be disclosed to the PI only, and the PI will report the SAE to the Ethics Committee so that a decision can be made regarding whether the subject needs to withdraw from the trial. All unexpected and unintended adverse events (AEs) will be recorded at each visit. During the screening and follow-up (week 6) periods, every participants will undergo blood tests, including liver function, blood glucose, and kidney function; every female participant will also receive urine pregnancy tests.

### Blinding assessment

Allocation guessing about the real TEA group vs. the sham TEA group will be assessed after the first treatment and after the last treatment. The subjects will select one of the following three items on a questionnaire about their allocation guess: “classical acupuncture typically used in Korean Medicine clinics,” “non-classical acupuncture, rarely used in Korean Medicine clinics,” or “don’t know” [58].

### Statistical methods

#### Sample size

This pilot RCT is designed as a preliminary to a full-scale confirmatory trial. Therefore, we assume that a total of 50 participants will be an acceptable sample size, considering 20 participants in each group with a 20% drop-out rate.

#### Statistical analysis

Data will be analyzed by an independent statistician who is blinded to the group allocation and who will use the Statistical Package for the Social Sciences (SPSS) Version 23.0 (IBM Inc., New York, NY, USA). The analysis set will consist of the Intent-to-treat (ITT) set, Per-protocol (PP) set, and Full Analysis Set (FAS). The ITT set will include all participants as originally allocated after randomization. The PP set will include the participants who completed three or more treatment sessions (75%) and the entire trial without any major violation of the protocol. Participants who are assessed at least once after random allocation will compose the FAS, which will be used for the main analysis of the treatment outcomes. Safety will be analyzed using the ITT set. The significance level will be set at *p* < 0.05. Missing data will be handled using the last-observation-carried-forward (LOCF) method.

The demographic and baseline characteristics, such as age, gender, medical history, duration of CNP and treatment expectancy, will be summarized for each treatment group. Depending on whether a normal distribution is found or not, baseline assessments for homogeneity will be conducted using the Wilcoxon rank sum test or the *t* test for continuous variables and Fisher’s exact test or the chi-square test for categorical variables.

The primary outcome, the mean changes from baseline to week 6 in the VAS scores, will be compared between the two groups by using the *t* test or analysis of covariance (ANCOVA). If the baseline difference in the VAS score between the two groups is statistically significant, an ANCOVA will be performed with the baseline VAS score as a covariate and the study group as a fixed factor. The paired *t* test or the Wilcoxon signed rank test will be conducted to compare the treatment outcomes before and after intervention in each group. In addition, a repeated-measures analysis of variance (ANOVA) will be used to further confirm the differences in the trends of the visit. The secondary efficacy outcomes will be evaluated using the Wilcoxon rank sum test or the *t* test for continuous variables and Fisher’s exact test or the chi-square test for categorical variables. For safety assessments, all adverse events will be recorded as frequencies with percentages and durations. The chi-square test or Fisher’s exact test will be used to evaluate the statistical significance of between-group differences in the frequencies of adverse events. The duration of each adverse event will be compared between the two groups by using the Wilcoxon rank sum test or the *t* test. Subgroup analyses based on baseline characteristics will also be performed for the primary and the secondary outcomes.

### Quality control

All researchers will undergo special training on recruitment, screening, randomization, assessment, instructions for completing the case report form (CRF), etc., for the purpose of conducting a high-quality clinical trial. Simulations based on the role of each researcher as specified in the pre-defined SOP will also be performed. A qualified clinical research associate (CRA) who is blinded to the subjects’ assignment will regularly monitor the overall progress of the on-going research, such as the completion of informed consent forms, the screening process according to the protocol, the recording of adverse events, and the recording and management of data on the CRF. The monitoring process, which is independent from investigator and sponsor, will consist of four phases: a site initiation visit, initiative monitoring visit at first subject enrollment, interim monitoring visits during the clinical trial, and a close-out visit upon completion of clinical trial.

## Discussion

TEA is a new type of acupuncture that can extend the therapeutic stimulation through the use of thread embedded at certain acupoints. Several case reports on the effectiveness of PDO TEA have been published [29, 59–61], but, to the best of our knowledge, no RCT investigating the efficacy of the embedded PDO thread in treating patients with CNP has been reported. Therefore, in this trial, we will designate a sham TEA group as a control group to evaluate the differences in the therapeutic effects caused by the PDO thread.

At present, a practitioner-patient-blinded (double-blinded) sham TEA device has been neither developed nor validated. Therefore, we will use a hollow TEA needle for sham TEA, and will also investigate whether the sham TEA is patient-blinded. In both groups, the hollow needle insertion in the TEA treatment will contribute to relieving neck pain due to the dry-needling effect [62, 63]. Nevertheless, we hypothesize that real PDO TEA will have a superior therapeutic effect because the participant will experience both the continuous stimulation caused by the embedded PDO thread and the initial stimulation caused by the dry-needling.

This study has some limitations. First, the follow-up period of 4 weeks is short. Second, the sample size (50 participants) is small. Third, the practitioner cannot be blinded to the allocation because of the absence of a double-blinded sham device for PDO TEA. Despite these limitations, this study is the first clinical trial to apply both PDO TEA and sham TEA in the treatment of patients with CNP.

The protocol has also been designed in accordance with the Standard Protocol Items: Recommendations for Intervention Trials (SPIRIT) 2013 (see Additional file 2) [64]. Every step of the trial will be rigorously conducted and monitored to ensure methodological integrity and scientific validity. The results of this study will provide valuable data and insights for a confirmative, full-scale RCT to determine the clinical effects of PDO TEA for the treatment of patients with CNP.

## Trial status

The recruitment of participants for this research began on 22 May 2017, and is presently ongoing. Recruitment is expected to be completed by the end of December 2017.

## Additional files

Additional file 1: Details of polydioxanone thread-embedding acupuncture treatments based on the Standards for Reporting Interventions in Clinical Trials of Acupuncture (STRICTA) Checklist 2010. (DOCX 28 kb)

Additional file 2: Standard Protocol Items; Recommendations for Interventional Trials (SPIRIT) 2013 Checklist: recommended items to address in a clinical trial protocol and related documents*. (DOCX 57 kb)

## Abbreviations

AE: Adverse event
ANCOVA: Analysis of covariance
ANOVA: Analysis of variance
CNP: Chronic neck pain
CRF: Case report form
DKMHDU: Dunsan Korean Medicine Hospital of Daejeon University
FAS: Full Analysis Set
HADS: Hospital Anxiety and Depression Scale
IRB: Institutional Review Board
ITT: Intent-to-treat
MCID: Minimal Clinically Important Difference
NDI: Neck Disability Index
NSAID: Non-steroidal anti-inflammatory drug
PDO: Polydioxanone
PGIC: Patient Global Impression of Change
PI: Primary investigator
PP: Per protocol
PPT: Pressure pain threshold
RCT: Randomized clinical trial
SAE: Serious adverse event
SOP: Standard operating procedures
TEA: Thread-embedding acupuncture
VAS: Visual analog scale
