# Supplementary material for: Efficacy and safety of polydioxanone thread embedded at specific acupoints for non-specific chronic neck pain: a study protocol for a randomized, subject-assessor-blinded, sham-controlled pilot trial
Source: Trials. 2018 Dec 6;19:672. doi: 10.1186/s13063-018-3058-9 (PMC6282385; doi:10.1186/s13063-018-3058-9)
Supplement: Supplementary file 1 — Details of polydioxanone thread-embedding acupuncture treatments based on the Standards for Reporting Interventions in Clinical Trials of Acupuncture (STRICTA) Checklist 2010. (DOCX 28 kb) [file 13063_2018_3058_MOESM1_ESM.docx]

**Additional file 1** Details of polydioxanone thread embedding acupuncture treatments based on the Standards for Reporting Interventions in Clinical Trials of Acupuncture (STRICTA) Checklist 2010.

| **Item** | **Detail** | **Description** |
| --- | --- | --- |
| **1. Acupuncture rationale** | 1a) Style of acupuncture (e.g. Traditional Chinese Medicine, Japanese, Korean, Western medical, Five Element, ear acupuncture, etc) | Polydioxanone(PDO) Thread embedding acupuncture (TEA) based on Complementary alternative medicine(CAM) |
|  | 1b) Reasoning for treatment provided, based on historical context, literature sources, and/or consensus methods, with references where appropriate | Textbook of Acupuncture and Moxibustion Medicine in Korean medicine [1], related studies [2-5] and expert consensus (Korean Medicine Doctor ; KMD) |
|  | 1c) Extent to which treatment was varied | Not applicable |
| **2. Details of needling** | 2a) Number of needle insertions per subject per session (mean and range where relevant) | A total of 14 TEA needles will be embedded at both sides. |
|  | 2b) Names (or location if no standard name) of points used (uni/bilateral) | The threads will be embedded bilaterally at local acupoints in the neck region. The seven TEA points used in this study are GB20, TE16, LI17, GB21, and SI14 for regular acupoints and two paravertebral points, one each at the levels of the fifth and the seventh cervical vertebrae (C5 and C7), for extra acupoints. |
|  | 2c) Depth of insertion, based on a specified unit of measurement, or on a particular tissue level | 1) Perpendicular insertion: 25mm, 38mm  2) Oblique insertion: in a shallow layer of muscle under the acupoint. |
|  | 2d) Response sought (e.g. *de qi* or muscle twitch response) | Not applicable  - Simple insertion technique without manipulation will be applied; a needle with an attached PDO thread is inserted to the depth at which the thread will be fully inserted, after which the needle is immediately removed. |
|  | 2e) Needle stimulation (e.g. manual, electrical) |  |
|  | 2f) Needle retention time |  |
|  | 2g) Needle type (diameter, length, and manufacturer or material) | - Disposable sterile PDO thread embedding devices (OV World Co., Seoul, Republic of Korea) with 29-gauge needles and USP size 6-0 PDO thread (Samyang Biopham Co., Seongnam-si, Republic of Korea) ​will be used  - Length: 1) 25-mm needle with 30-mm thread (15 mm x 2; folded in half) 2)38-mm needle with 54-mm thread (27 mm x 2; folded in half). |
| **3. Treatment regimen** | 3a) Number of treatment sessions | 4 times. |
|  | 3b) Frequency and duration of treatment sessions | A total of four TEA treatments will be provided once a week for 4 weeks |
| **4. Other components of treatment** | 4a) Details of other interventions administered to the acupuncture group (e.g. moxibustion, cupping, herbs, exercises, lifestyle advice) | - Co-intervention will not be allowed except rescue medicine (Acetaminophen, maximum dose 3,000mg / day). |
|  | 4b) Setting and context of treatment, including instructions to practitioners, and information and explanations to patients | - The Clinical Trial Center and the outpatient department of Dunsan Korean Medicine Hospital of Daejeon University  - The patients will not be allowed to expose their assigned group to the assessor, and the assessor will not be allowed to have conversations with the patients other than those necessary for the evaluation. The practitioner will not be allowed to have any conversations with patients other than those necessary for the appropriate TEA treatment, such as inquiry and palpation. |
| **5. Practitioner background** | 5) Description of participating acupuncturists (qualification or professional affiliation, years in acupuncture practice, other relevant experience) | The TEA and sham TEA will be performed by an individual Korean Medicine Doctor (KMD). The practitioner received six years of university education for Traditional Korean Medicine and has at least four years of clinical experience on TEA. |
| **6. Control or comparator interventions** | 6a) Rationale for the control or comparator in the context of the research question, with sources that justify this choice | - The sham TEA device will be a dry-needle with the PDO thread removed. The sham TEA device will be made by the same manufacturer and will be, for all practical purposes, identical to the real TEA device except for the absences of thread hanging outside the needle and the thread anchor. In other words, subjects in the control (sham TEA) group will receive only the stimulus of dry-needling, but not that of the embedded PDO thread. |
|  | 6b) Precise description of the control or comparator. If sham acupuncture or any other type of acupuncture-like control is used, provide details as for Items 1 to 3 above. | -Sham TEA will be performed in the same manner as the TEA and only difference between these two interventions is that PDO thread would not be embedded into the acupoints in the sham TEA group; a total of four TEA treatments will be provided once a week for 4 weeks |

1. Acupuncture Medicine. Seoul: Hanmi Medical Publishing Company; 2016.

2. Blossfeldt P. Acupuncture for chronic neck pain--a cohort study in an NHS pain clinic. Acupunct Med. 2004;22(3):146-51.

3. Kwon K. The Analysis on the Present Condition of Thread-embedding Therapy Papers Published in Journal of Korean Medicine. The Journal of Korean Medicine Ophthalmology and Otolaryngology and Dermatology. 2014;27(4):16-44.

4. Lee S, Nam D, Leem J, Han G, Lee S, Lee J. Efficacy and safety of Myofascial-meridian Release Acupuncture (MMRA) for chronic neck pain: a study protocol for randomized, patient- and assessor-blinded, sham controlled trial. BMC Complement Altern Med. 2016;16:45.

5. Liang ZH, Di Z, Jiang S, Xu SJ, Zhu XP, Fu WB, et al. The optimized acupuncture treatment for neck pain caused by cervical spondylosis: a study protocol of a multicentre randomized controlled trial. Trials. 2012;13:107.
